# WRKY Transcription Factors Modulate the Flavonoid Pathway of *Rhododendron chrysanthum* Pall. Under UV-B Stress

**DOI:** 10.3390/plants14010133

**Published:** 2025-01-04

**Authors:** Wang Yu, Xiangru Zhou, Jinhao Meng, Hongwei Xu, Xiaofu Zhou

**Affiliations:** Jilin Provincial Key Laboratory of Plant Resource Science and Green Production, Jilin Normal University, Siping 136000, China

**Keywords:** *Rhododendron chrysanthum* Pall., multi-omics, WRKY transcription factor, UV-B stress, metabolomics

## Abstract

The depletion of the ozone layer has resulted in elevated ultraviolet-B (UV-B) radiation levels, posing a significant risk to terrestrial plant growth. *Rhododendron chrysanthum* Pall. (*R. chrysanthum*), adapted to high-altitude and high-irradiation environments, has developed unique adaptive mechanisms. This study exposed *R. chrysanthum* to UV-B radiation for two days, with an 8 h daily treatment, utilizing metabolomic and transcriptomic analyses to explore the role of WRKY transcription factors in the plant’s UV-B stress response and their regulation of flavonoid synthesis. UV-B stress resulted in a significant decrease in rETR and Ik and a significant increase in 1-qP. These chlorophyll fluorescence parameters indicate that UV-B stress impaired photosynthesis in *R. chrysanthum*. Faced with the detrimental impact of UV-B radiation, *R. chrysanthum* is capable of mitigating its effects by modulating its flavonoid biosynthetic pathways to adapt positively to the stress. This study revealed changes in the expression of 113 flavonoid-related metabolites and 42 associated genes, with WRKY transcription factors showing significant correlation with these alterations. WRKY transcription factors can influence the expression of key enzyme genes in the flavonoid metabolic pathway, thereby affecting metabolite production. A theoretical reference for investigating plant stress physiology is provided in this work, which also offers insights into the stress responses of alpine plants under adverse conditions.

## 1. Introduction

The Earth’s surface is exposed to ultraviolet (UV) radiation, which originates from sunlight and has a significant impact on ecosystems [1]. Ultraviolet radiation can be categorized into three main bands, i.e., long wave (315–400 nm, UV-A), medium wave (280–315 nm, UV-B), and short wave (200–280 nm, UV-C), with UV-B having the most widespread impact on living organisms in nature [2,3,4]. Ultraviolet-B (UV-B) stress is a common abiotic factor that can severely limit plant growth and development [5]. UV-B radiation breaks down the structure of macromolecular DNA, alter plant morphology, reduce the plant photosynthetic rate, and affect plant productivity [6,7,8].

Transcriptomic studies on peach peel following UV-B stress indicate that UV RESISTANCE LOCUS 8 (*UVR8*) may be involved in phenolic biosynthesis [9]. Phosphorylated proteomic studies have shown that UV-B radiation can activate oxidative stress response, photosynthetic energy metabolism, and MAPK signal transduction and promote the accumulation of secondary metabolites [10]. Metabolomic and transcriptomic studies have shown that amino acids and carbohydrates promote resistance to UV-B stress in *R. chrysanthum* [11]. Studies have shown that the secondary metabolites (tocopherols, polyamines, and flavonoids) of plants have different accumulation patterns under UV-B domestication [12]. *UVR8* is involved in regulating plant growth and development as a photoreceptor. Studies on *UVR8* in apple showed that *UVR8* plays an important role in regulating photomorphogenesis under UV-B light [13]. Hydrogen peroxide, as a signaling molecule, can mediate the synthesis of flavonoids under UV-B radiation [14]. It has been shown that plants are able to protect themselves against UV-B radiation damage by inducing the synthesis of UV-absorbing substances such as flavonoids [15,16]. Flavonols in Ginkgo biloba, especially isorhamnetin, increased exponentially in response to in response to UV-B stress [17]. Flavonoid synthases in maize are involved in the synthesis of flavonoids such as apigenin against UV-B stress [18]. Understanding the molecular mechanisms of plant resistance to UV-B radiation is beneficial for screening varieties with strong stress resistance [19].

As sessile organisms, plants cannot move like an animal to escape their environmental conditions. Plants adapt to harsh environments by adjusting the content of various metabolites and altering their developmental processes [20]. *R. chrysanthum* is an important plant resource. It grows at an altitude of 1300–2650 m in the Changbai Mountains in China. It is cold there all year round, and plants are exposed to strong UV radiation and strong winds. During evolution, *R. chrysanthum* was able to adapt to this extreme environment [21]. There are many studies on plant cold resistance that have found that the antioxidant system of *R. chrysanthum* is enhanced during long-term acclimatization [22]. Quantitative proteomic and phosphorylated proteomic results indicate that multiple-reactive oxygen species clearance and calcium-mediated signaling pathways are triggered to resist cold stress [23]. In a previous study, the combination of 2.3 W·m^−2^ and 2 days of radiation treatment was determined to have the most significant negative effect on the fluorescence characteristics of *R. chrysanthum* by varying the intensity of UV-B radiation with the duration of radiation [24].

However, less research has been performed on how alpine plants resist against UV-B stress. Metabolomic studies have shown that the accumulation of flavonoids, organic acids, amino acids, and fatty acids may protect *R. chrysanthum* from UV-B harm [21]. Proteomic studies have shown that UV-B stress starts the photosynthesis pathway in plants and photosystem II (PSII) proteins are possibly acetylated to reduce impairment [25]. The long-term acclimatization of *R. chrysanthum* helps to study its reaction to UV-B stress. Studies have confirmed that *R. chrysanthum* is sensitive to UV-B radiation and exhibits effective antagonistic mechanisms [24]. Therefore, *R. chrysanthum* is a good material for studying UV-B stress.

WRKY TFs are associated with the adjustment of plants in response to abiotic stress. Studies on *Nicotiana benthamiana* showed that *GhWRKY41* could act as a positive regulator of stomatal closure, the modulation of reactive oxygen species (ROS) scavenging, and the expression of antioxidant genes to enhance plant tolerance to salt stress and drought stress [26]. Studies on WRKY transcription factors in soybean showed that *GmWRKY21* was able to regulate tolerance to cold stress in transgenic *Arabidopsis* plants, whereas *GmWRKY54* may confer salt and drought tolerance to *Arabidopsis* by regulating DREB2A and STZ/Zat10 [27]. Investigating the alterations in WRKY TFs within the leaves of *R. chrysanthum* under UV-B stress may enhance our understanding of the molecular mechanisms underlying UV-B radiation tolerance in this species.

Widely targeted metabolomics, which can detect more metabolites, is the latest technology in metabolomic research [28]. A study was conducted to characterize phenolic compounds by using UPLC-MS/MS to study the variation in the content of polyphenols in *Spondias mombin* L. [29]. The metabolite profiles of *R. chrysanthum* in the M group (normal light) and the N group (normal light +UV-B radiation) were analyzed and quantitatively compared based on UPLC-MS/MS. See Section 4 for specific material treatments.

In a broader application of transcriptomics, some researchers have used transcriptomics to study the role of UV-B dose in regulating L-ascorbic acid (AsA) biosynthesis in *Lactuca sativa* [30]. Examining the transcriptome of peach skins vacuum freeze-drying UV-B exposure revealed the activation of genes involved in phenolic biosynthesis, informing the use of UV-B treatments to increase the content of health-promoting compounds in peach fruit [9]. Transcriptomics was used to study the molecular mechanisms of peach trees under low-temperature stress, and it was found that soluble sugar, flavonoid, and lignin biosynthesis-related genes may play important roles in cold tolerance in peach [31]. Numerous studies have shown that transcriptomics plays an important role in the study of plant response to abiotic stresses.

Joint analysis of transcriptomics and metabolomics is an important method for omics research. One study utilized this approach to investigate the effects of short-term CO_2_ treatment on the postharvest quality of chili peppers and the molecular mechanism of their regulation, which provides a reference for the study of crop preservation conditions [32]. The study of post-irradiation mouse blood using combined transcriptomic and metabolomic analyses has made a significant contribution to the field of biomedicine by finding that this approach helps to provide a deeper understanding of the crosstalk between different biomolecules [33]. Metabolomic and transcriptomic association analyses can study key pathways involving genes in differential metabolite synthesis and understand how key substances are regulated following UV-B radiation.

In order to investigate the plant response to UV-B stress, both normal light- and UV-B-irradiated *R. chrysanthum* specimens were utilized in this study. We examined the metabolites and associated genes that exhibited significant alterations in the leaves of *R. chrysanthum* under UV-B radiation conditions through metabolomic and transcriptomic analyses. Additionally, we analyzed the TFs that may be involved in flavonoid synthesis. This research study provides a valuable reference for understanding how flavonoids respond to UV-B stress and enhances our comprehension of their antagonistic effects.

## 2. Results

### 2.1. Significant Changes in Chlorophyll Fluorescence Parameters Occur Following UV-B Treatment

**Figure 1:**
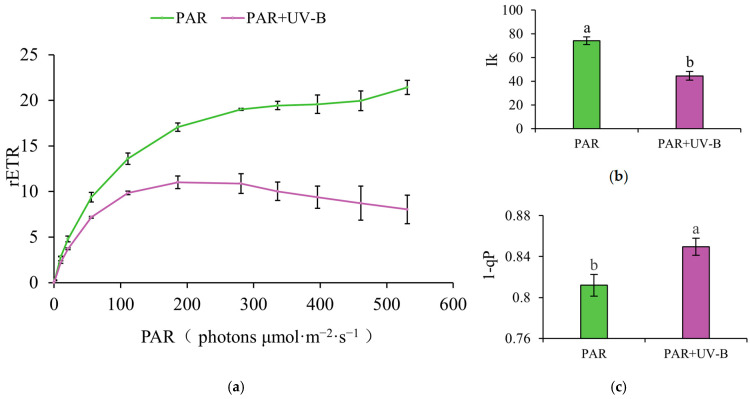
Changes in chlorophyll fluorescence parameters following UV-B radiation. (**a**) Line graph of rETR; (**b**) bar graph of I_K_; (**c**) bar graph of “1-qP”. rETR can estimate the electron transfer rate of PSII under specific light conditions, reflecting the photosynthetic potential of plants. Ik can reflect the plant’s ability to tolerate strong light; the higher Ik is, the stronger the sample’s tolerance to strong light is. The “1-qP” represents the reduced state of QA, which can reflect the degree of photosystem II shutdown. The bar graph illustrates the mean heights for each group, based on three biological replicates (*n* = 3). Error bars represent the standard deviations among these replicates. Groups marked with different letters were significantly different from each other (*p* < 0.05), indicating statistically significant variations.

Following exposure to environmental stress, alterations in the photosynthetic process of plants can be reflected in the parameters of chlorophyll fluorescence. Chlorophyll fluorescence metrics that were pertinent to the UV-B-treated study subjects were established in this investigation. Following exposure to UV-B light, it became obvious that *R. chrysanthum*’s photosystem II of rETR (relative electron transfer rate) dramatically dropped (Figure 1a). The plant’s capacity to withstand intense light is indicated by “I_k_”, while the percentage of photosystem II reaction centers in the off state is shown by “1-qP”. UV-B caused a significant decrease in I_K_ and a significant increase in “1-qP” (Figure 1b,c). It could mean that UV-B stress severely restricted *R. chrysanthum*’s ability to carry out photosynthesis ordinarily. For the purpose of safeguarding itself from UV-B damage, *R. chrysanthum* initiates its own metabolic defense system.

### 2.2. UV-B Radiation Significantly Alters Flavonoid Content in Leaves of R. chrysanthum

To study the effect of UV-B radiation on the leaves of *R. chrysanthum*, we characterized the metabolites in the M and N groups. The PCA of the identified metabolites revealed that the M and N groups were clearly distinguished, and three replicates of each group were clustered together, indicating good intragroup reproducibility and significant differences between the groups (Figure S1a). To further identify the metabolites associated with leaf resistance against UV-B radiation, we used OPLS-DA analysis to explore differential metabolites between the M and N groups (Figure S1b). The first principal component explained 28.9% of the dataset, and the second principal component explained 28.3% of the dataset, giving reliable results. In this study, the screening criteria for differential metabolites were set as follows: FC (fold change) ≥ 1.5, FC ≤ 0.67, and VIP > 1. As shown in Figure S1c, the contents of 355 metabolites were increased, and 167 metabolites were decreased following UV-B radiation. These 522 differential metabolites were ranked according to FC, and the top 30 metabolites (15 metabolites with elevated levels and 15 metabolites with reduced levels) are shown in Figure S1d.

**Figure 2:**
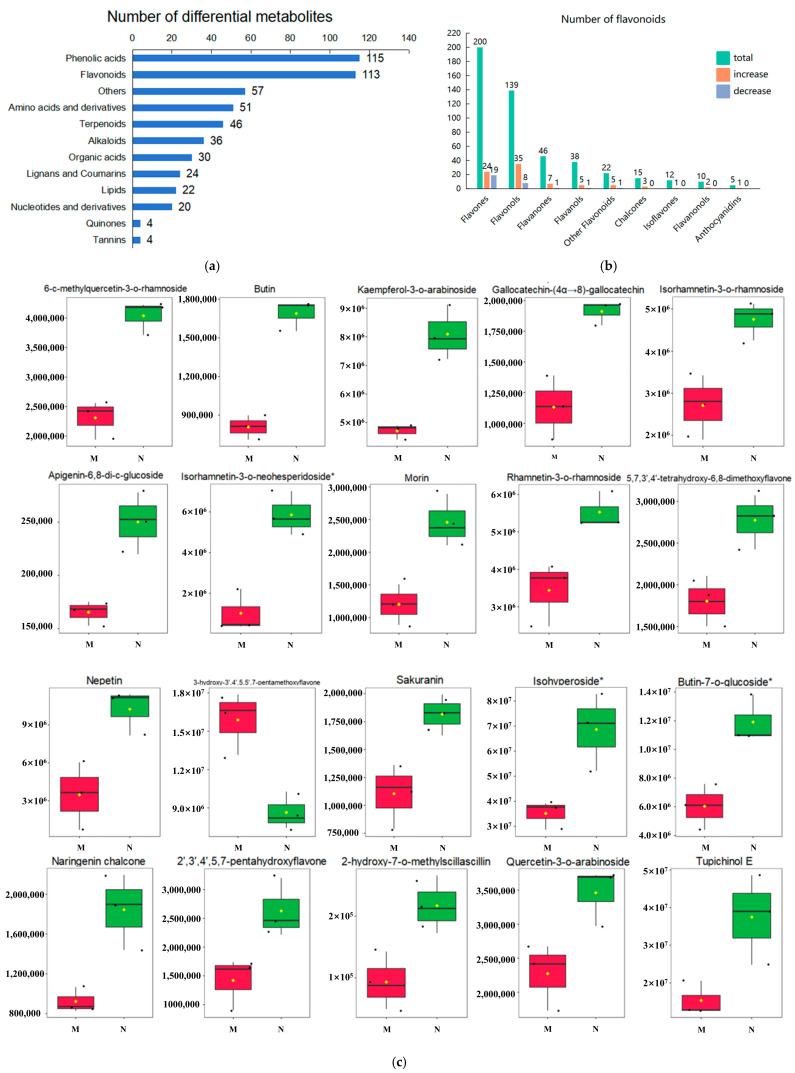

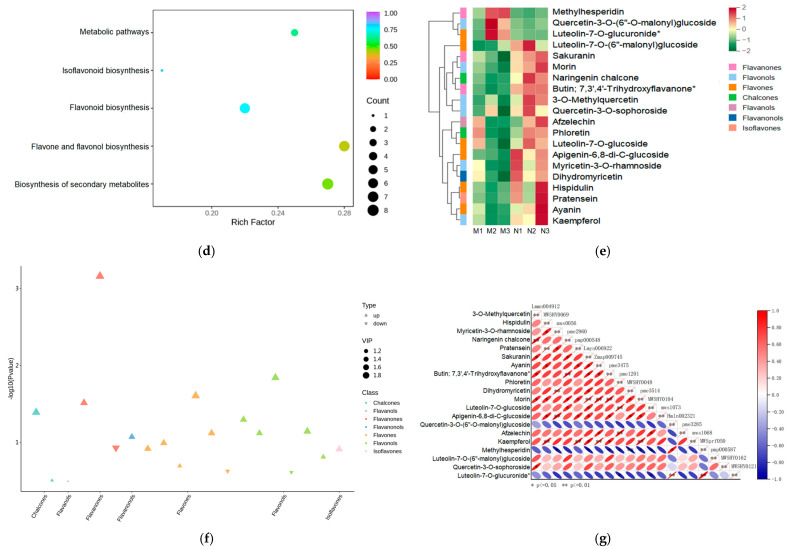
Significant changes in flavonoids in *R. chrysanthum* following UV-B stress. (**a**) Differential metabolite classification statistical chart; (**b**) statistical chart of secondary classification of flavonoids; (**c**) contents of top 20 metabolites of VIP value in flavonoids; (**d**) bubble map of KEGG enrichment analysis of flavonoids; (**e**) heat map of clustering of flavonoids with significant differences; (**f**) scatterplot of differential flavonoids able to be annotated to KEGG database; (**g**) correlation analysis of significantly different flavonoids. In the bubble plot, the bubble size represents the number of differential flavonoids. The bubble color represents the size of the corresponding pathway enrichment significance. In the heatmap, redder colors indicate higher metabolite content, and greener colors indicate lower content. “*” indicates that *p* is less than or equal to 0.05, indicating a strong correlation between the two metabolites; “**” indicates that *p* is less than or equal to 0.01, indicating an extremely strong correlation between the two metabolites.

Our categorical statistics of the differential metabolites obtained from the screening revealed that the amounts of phenolic acids (115) and flavonoids (113) were the highest following UV-B stress (Figure 2a). In addition, amino acids and derivatives (51), terpenoids (46), alkaloids (36), and organic acids (30) also changed significantly following UV-B stress, which may play an influential role in the resistance of *R. chrysanthum* to UV-B stress.

We explored the response of phenolic acids in *R. chrysanthum* to UV-B stress in a previous study, so the focus of this paper is on flavonoids [34]. We performed a quantitative count of the secondary classification of flavonoids, and the results are shown in Figure 2b. There were 113 flavonoids among the screened differential metabolites, of which 83 flavonoids were increased and 30 flavonoids were decreased following UV-B radiation. These 113 flavonoids can be categorized into nine groups, with “flavones” and “flavonols” having the most, with 43 each; “flavanones” having 8; “flavanols” and “other flavonoids” having 6 each; “chalcones” having 3; “flavanonols” having 2; and “anthocyanidins” and “isoflavones” having 1 each.

The top 20 metabolites of the differential flavonoid VIP are illustrated in Figure S1e, and the box plot in Figure 2c shows the contents of these 20 flavonoids. The cumulative pattern of 3-hydroxy-3′,4′,5,5′,7-pentamethoxyflavone among these 20 metabolites was different from that of the other metabolites, suggesting that 3-hydroxy-3′,4′,5,5′,7-pentamethoxyflavone may be a negatively responsive metabolite to UV-B radiation in *R. chrysanthum*. The KEGG enrichment analysis of differential flavonoids was performed, and the bubble plots show that these 113 metabolites were mainly enriched in five metabolic pathways: “metabolic pathways”, “isoflavonoid biosynthesis”, “flavonoid biosynthesis”, “flavone and flavonol biosynthesis”, and “biosynthesis of secondary metabolites” (Figure 2d). The number and proportion of differential metabolites annotated to these five metabolic pathways are shown in the KEGG classification diagram (Figure S1f). A total of 20 of these 113 significantly different flavonoids were annotated to the KEGG database, and the contents of these 20 metabolites were higher for 17 and lower for 3 (“methylhesperidin”, “quercetin-3-o-(6″-o-malonyl) glucoside”, and “luteolin-7-o-glucuronide*”), as shown in the heat map (Figure 2e). Of these 20 differential flavonoids that were able to be annotated to the KEGG database, flavones and flavonols, six of each, accounted for 30% of the total (Figure 2f).

To understand the relationships among these 20 metabolites, a correlation analysis was performed (Figure 2g). Naringenin chalcone and 3-o-methylquercetin were significantly positively correlated. Pratensein and hispidulin were significantly positively correlated. Butin and naringenin chalcone were significantly positively correlated. Dihydromyricetin was significantly positively correlated with myricetin-3-o-rhamnoside. Morin was significantly positively correlated with sakuranin, ayanin, and butin. Luteolin-7-o-glucoside was significantly positively correlated with phloretin. Apigenin-6, 8-di-c-glucoside and myricetin-3-o-rhamnoside were significantly positively correlated. Afzelechin and phloretin were significantly positively correlated. Kaempferol was positively correlated with hispidulin, pratensein, ayanin, and morin. Methylhesperidinl and dihydromyricetin were significantly negatively correlated. Luteolin-7-o-glucuronide*, and quercetin-3-o-(6″-o-malonyl) glucoside, methylhesperidin were significantly positively correlated. The metabolites identified by widely targeted metabolomics are shown in Table S1.

### 2.3. UV-B Radiation Significantly Alters Flavonoid-Related Genes in Leaves of R. chrysanthum

**Figure 3:**
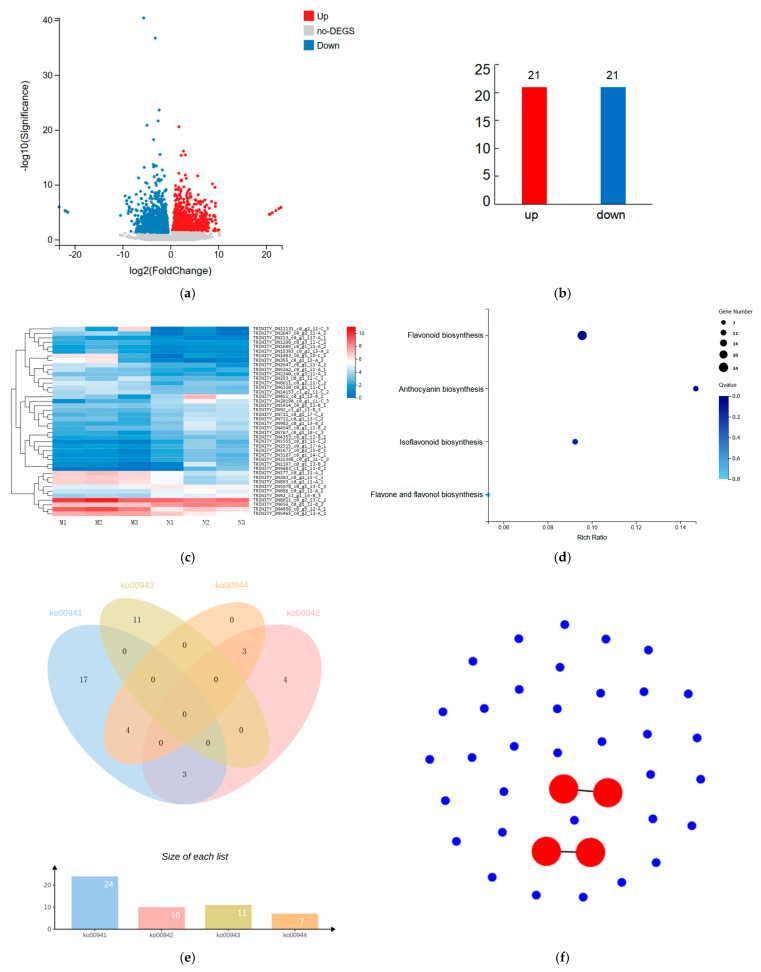
Transcriptomic data showed significant changes in genes related to flavonoids following UV-B radiation. (**a**) Volcano map of DEGs; (**b**) statistics on number of DEGs related to flavonoids; (**c**) heat map of DEGs related to flavonoids following UV-B stress; (**d**) KEGG bubble chart; (**e**) Venn diagram of differentially expressed genes; (**f**) protein–protein interaction network map of genes associated with flavonoid synthesis. In the bubble plot, the bubble size represents the number of DEGs. The bubble color represents the corresponding pathway enrichment significance size.

We performed transcriptomic assays on M and N group *R. chrysanthum* leaves for genes that respond to UV-B radiation. The transcriptomic data showed that 66,659 genes were expressed simultaneously in the M and N groups, 4137 genes were expressed only in the M group and 4763 genes were expressed only in the N group. Q-value < 0.05 and FC > 1 were used as the screening criteria for DEGs between the M and N groups. The results of DEG screening are shown in the volcano diagram, with a total of 2348 DEGs, of which 1157 were up-regulated and 1191 were down-regulated (Figure 3a). Of the 2348 DEGs, 42 were associated with flavonoids, with 21 being up-regulated and 21 being down-regulated (Figure 3b). The expression information of 42 genes is shown in a heat map (Figure 3c).

The KEGG enrichment analysis of the differentially expressed genes revealed enrichment in four pathways: flavonoid biosynthesis (24), anthocyanin biosynthesis (10), isoflavonoid biosynthesis (11), and flavone and flavonol biosynthesis (7) (Figure 3d). We counted the annotation of 42 DEGs in four pathways and found that 10 genes were able to be annotated in two pathways at the same time (Figure 3e). The KEGG enrichment analysis bar graph shows that these 42 genes are involved in two branches of the KEGG metabolic pathway: environmental information processing and metabolism (Figure S2a). The most enriched entry in the GO enrichment histogram is UDP-glycosyltransferase activity (9) (Figure S2b). These genes can be categorized into three main groups according to their functions: molecular function, cellular component, and biological process. Twenty-five of them had catalytic activity, nine had cellular anatomical entity, and eight had binding (Figure S2c). By performing protein–protein interaction (PPI) networks on these 42 genes, it was found that TRINITY_DN8611_c0_g2_i1-C_2 and TRINITY_DN8611_c0_g2_i3-C_2 were highly correlated and RINITY_DN21396_c0_g1_i1-C_3 and TRINITY_DN4856_c0_g5_i2-A_1 were highly correlated (Figure 3f). Information on the 42 DEGs associated with flavonoids is shown in Table S2.

### 2.4. Differential Flavonoids Are Strongly Correlated with Differentially Expressed Genes

In order to understand the relationship between differentially significant flavonoids and genes, transcriptomic and metabolomic correlation analyses were performed. We constructed a correlation network graph for 113 significantly different flavonoids and genes and found that there were 363 edges in the graph, with 196 edges indicating positive correlations and 167 edges indicating negative correlations. The largest number of genes (22) were correlated with butin, with 11 positive and 11 negative correlations. There were 21 genes associated with naringenin chalcone, 14 positively and 7 negatively.

**Figure 4:**
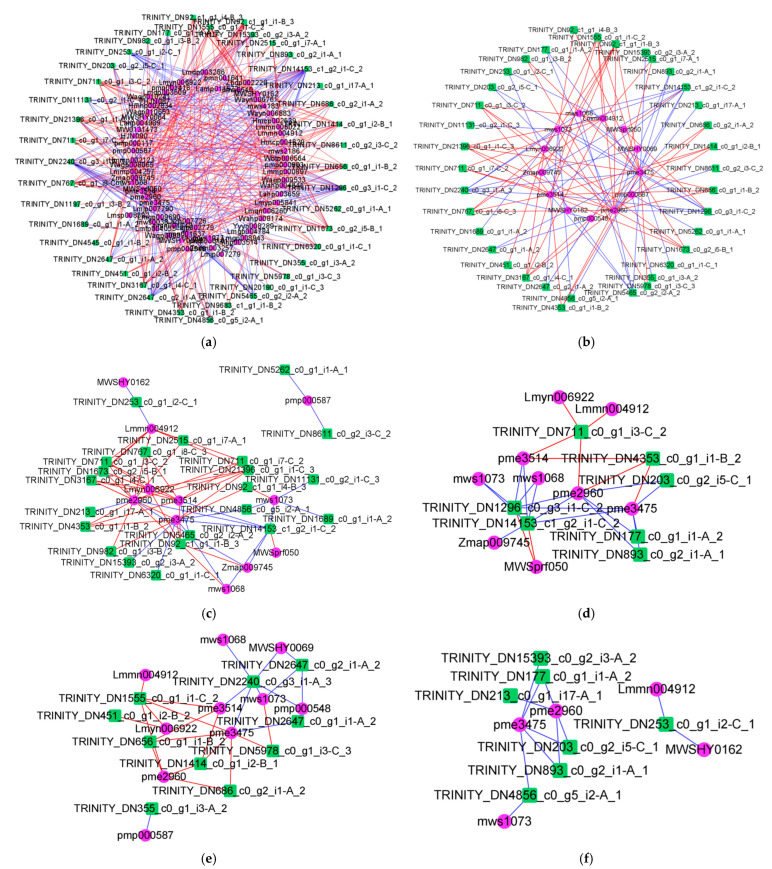
Transcriptomic and metabolomic analysis. (**a**) Analysis of 113 flavonoid metabolites with genetic correlation; (**b**) analysis of 20 flavonoid metabolites with genetic correlation; (**c**–**f**) correlation analysis of metabolites with ko00941 pathway-related genes, ko00942 pathway-related genes, ko00943 pathway-related genes, and ko00944 pathway-related genes, respectively. When using the Pearson correlation analysis method, the correlation analysis threshold was 0.9, *p* < 0.05. Pink nodes indicate metabolites, and green nodes indicate genes; red edges indicate positive correlations, and blue edges indicate negative correlations.

Metabolites with more than 15 edges were also gallocatechin-(4α->8)-catechin-(4α->8)-catechin (19), kaempferol-3-o-arabinoside (18), 6-c-methylquercetin-3-o-rhamnoside (18), and 3-hydroxy-3′,4′,5,5′,7-pentamethoxyflavone (18) (Figure 4a).

The 20 metabolites that could be annotated to the KEGG database were analyzed for correlation with the genes, and the results are shown in Figure 4b, with a total of 103 edges, with 58 indicating positive correlations and 45 indicating negative correlations. The edges linked to butin (increased content) and naringenin chalcone (increased content) were 22 and 21, respectively. Since these 20 metabolites annotated to the KEGG database were enriched in four pathways, we investigated the correlation between metabolites and genes in the four pathways. As shown in Figure 4c–f, there were 63 edges in the flavonoid biosynthesis pathway (41 positive correlations and 22 negative correlations) and 26 edges in the anthocyanin biosynthesis pathway (9 positive correlations and 17 negative correlations). There were 28 edges in the isoflavonoid biosynthesis pathway (16 positive and 12 negative) and 12 edges in the flavone and flavonol biosynthesis pathway (0 positive and 12 negative). Among these four pathways, genes related to butin, naringenin chalcone, and morin were all more abundant, suggesting that these three metabolites may be important for *R. chrysanthum* to resist against UV-B stress.

### 2.5. WRKY Transcription Factors Play a Crucial Role in UV-B Stress Resistance in R. chrysanthum

**Figure 5:**
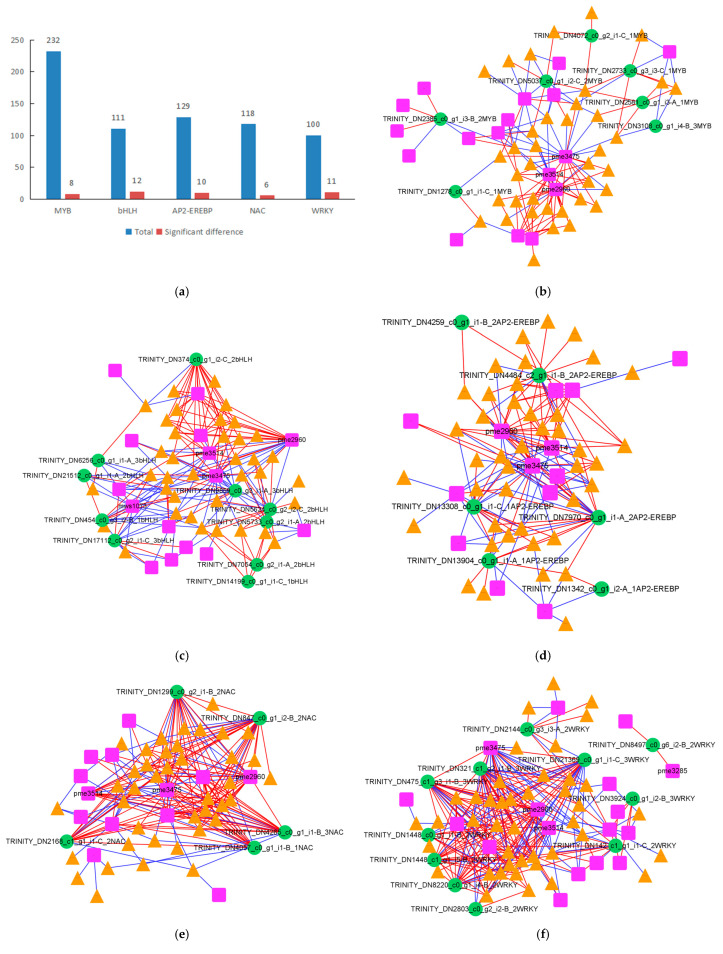
WRKY TFs play an important role in *R. chrysanthum*’s resistance to UV-B stress. (**a**) Number of transcription factors statistics; (**b**) correlation analysis involving MYB transcription factors; (**c**) correlation analysis involving bHLH transcription factors; (**d**) correlation analysis involving AP2-EREBP transcription factors; (**e**) correlation analysis involving NAC transcription factors; (**f**) correlation analysis involving WRKY transcription factors. In the bar chart, blue indicates the number of genes capable of encoding transcription factors detected in *R. chrysanthum*, and red indicates the number of genes with the ability to encode transcription factors that differed significantly between the M and N groups. When using the Pearson correlation analysis method, the correlation analysis threshold was 0.9, *p* < 0.05. Pink boxes indicate metabolites, green circles indicate genes capable of encoding TFs, and orange triangles indicate DEGs. Red edges indicate positive correlations, and blue edges indicate negative correlations.

Gene expression is regulated by transcription factors (TFs), and in order to explore the changes in the flavonoid-related pathways of *R. chrysanthum* following UV-B stress, we counted the DEGs identified as TFs. The number of transcription factors (TFs) potentially related to flavonoids is shown in Figure 5a and involves five families of transcription factors, namely, MYB, bHLH, AP2-EREBP, NAC, and WRKY.

Two hundred and thirty-two genes were identified as members of the MYB TF family, eight of which were significantly different following UV-B stress. One hundred and eleven genes were identified as members of the bHLH TF family, twelve of which were significantly different following UV-B stress. One hundred and twenty-nine genes were identified as members of the AP2-EREBP family of TFs, ten of which were significantly different following UV-B stress. One hundred and eighteen genes were identified as members of the NAC TF family, six of which were significantly different following UV-B stress. One hundred genes were identified as members of the WRKY TF family, eleven of which were significantly different following UV-B stress.

In order to systematically identify unknown TFs controlling flavonoid synthesis in the M and N groups of *R. chrysanthum* leaves following UV-B stress, correlation analyses were performed for differentially significant flavonoids, DEGs, and differentially significant TFs. The correlation of flavonoids and DEGs capable of being annotated to the KEGG pathway with these five families of TFs is illustrated (Figure 5b–f). Figure 5b–f each have 137, 198, 165, 207, and 262 edges, indicating strong correlations among the screened genes, TFs, and metabolites, which may be related to flavonoid regulation. The strong correlation of each TF family with 3-o-methylquercetin, naringenin chalcone, butin, morin, and apigenin-6,8-di-C-glucoside suggests that these five metabolites are the key metabolites regulated by TFs.

Correlation analysis involving MYB TF families revealed that 7 flavonoids and 16 DEGs were strongly correlated with 7 MYB TFs, respectively, with 7 edges being related to flavonoids and 27 edges being related to DEGs. Correlation analysis involving bHLH TF families revealed that 13 flavonoids and 35 DEGs were strongly correlated with 10 bHLH TFs, respectively, with 23 edges being related to flavonoids and 72 edges being related to DEGs. Correlation analysis involving the AP2-EREBP TF family revealed that 2 flavonoids and 35 DEGs were strongly correlated with each of the 6 AP2-EREBP TFs, with 4 edges being associated with flavonoids and 58 edges being associated with DEGs. Correlation analysis involving the NAC TF families revealed that 6 flavonoids and 33 DEGs were strongly correlated with each of the 5 NAC TFs, with 15 edges being related to flavonoids and 89 edges being related to DEGs. Correlation analysis involving WRKY TF families revealed that 13 flavonoids and 36 DEGs were strongly correlated with 11 WRKY TFs each, with 26 edges being related to flavonoids and 133 edges being related to DEGs. The correlation analyses of TFs with differentially significant flavonoids and DEGs revealed that WRKY TFs play an important role in the resistance of *R. chrysanthum* against UV-B stress (Figure 5b–f).

### 2.6. WRKY TFs Regulate Expression of Key Enzyme Genes in Flavonoid Synthesis Pathway

**Figure 6:**
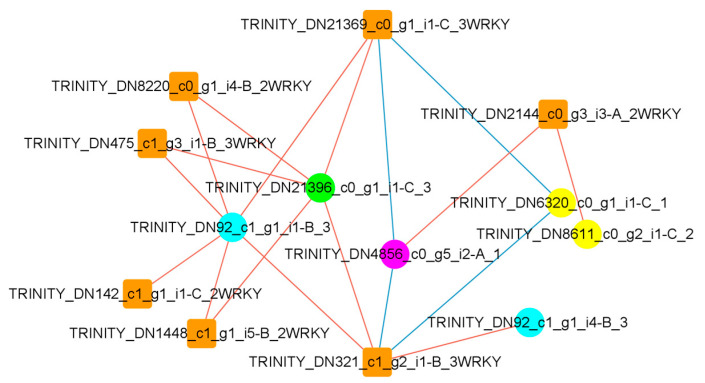
WRKY TFs are strongly correlated with key enzymes in the flavonoid synthesis pathway. When using the Pearson correlation analysis method, the correlation analysis threshold was 0.9, *p* < 0.05. Boxes indicate WRKY TFs, and circles indicate genes encoding 4 enzymes, where pink: flavonoid 3′,5′-hydroxylase; blue: chalcone isomerase; green: dihydromyricetin reductase; yellow: flavonol synthase. The red (blue) edges represent positive (negative) correlations.

The KEGG database was utilized to localize the DEGs into flavonoid-related pathways, and it was found that the DEGs were able to encode four enzymes, namely, 3′,5′-hydroxylase, chalcone isomerase, dihydromyricetin reductase, and flavonol synthase. Information on the genes encoding the enzymes is shown in Table 1. We correlated the WRKY TFs that changed significantly following UV-B stress with the genes encoding these four enzymes (Figure 6) and found that three WRKY TFs were strongly correlated with flavonoid 3′,5′-hydroxylase (one positively and two negatively), six WRKY TFs were significantly positively correlated with chalcone isomerase, five WRKY TFs were significantly positively correlated with dihydromyricetin reductase, and three WRKY TFs were strongly correlated with flavonol synthase (one positively and two negatively).

In order to better understand the relations between differential metabolites and DEGs, we combined flavonoids annotated in the KEGG database with DEGs to construct a network to gain a clearer insight into the interrelationships between gene expression and flavonoid accumulation in *R. chrysanthum* leaves under UV-B stress.

**Figure 7:**
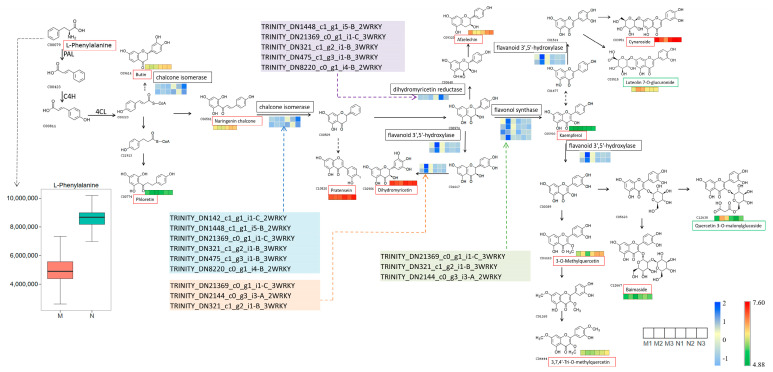
WRKY TFs can regulate the expression of key enzyme genes in the flavonoid synthesis pathway to improve tolerance to UV-B stress in *R. chrysanthum*. For the DEG heatmap, we used the z-score algorithm, and for the metabolite heatmap, we used substance content. A red box around a metabolite indicates that the metabolite was elevated following UV-B radiation, and a green box indicates that the level was reduced. Names in the colored boxes indicate transcription factors associated with flavonoids.

As shown in the box-and-line diagram in Figure 7, L-phenylalanine expression was elevated following UV-B stress, leading to the accumulation of downstream flavonoids. As can be seen in Figure 7, the contents of phloretin, naringenin chalcone, butin, dihydromyricetin, kaempferol, and afzelechin, annotated to the flavonoid biosynthesis pathway, were significantly elevated in N group *R. chrysanthum*. The contents of pratensein levels annotated to the isoflavonoid biosynthesis pathway were significantly higher, and luteolin-7-o-glucoside, ayanin, 3-o- methylquercetin, quercetin-3-o-sophoroside, and kaempferol levels were significantly elevated, while luteolin-7-o-glucuronide* and quercetin-3-o-(6″-o-malonyl) glucoside levels were significantly reduced. The genes encoding the WRKY TFs in the orange box may affect the accumulation of taxifolin, luteolin, quercetin, and dihydromyricetin by affecting the genes encoding 3′,5′-hydroxylase. The genes encoding the WRKY TFs in the blue box may influence the accumulation of naringenin and butin by affecting the genes encoding chalcone isomerase. The genes encoding the WRKY TFs in the purple box may influence cis-3,4-leucopelargonidin accumulation by affecting the gene encoding dihydromyricetin reductase. The genes encoding WRKY TFs in the green box may influence kaempferol accumulation by affecting the genes encoding flavonol synthase.

## 3. Discussion

UV-B radiation affects plant growth as abiotic stress due to the degradation of the ozone layer, which enhances the UV-B radiation reaching the plant surface [35]. UV-B radiation is capable of causing damage to the photosynthetic apparatus of green plants at multiple locations [36]. *R. chrysanthum* is a plant resource that grows in alpine environments and is exposed to intense UV-B radiation while growing. Previous experiments have demonstrated that the leaves of *R. chrysanthum* are able to respond to UV-B stress, mainly in photosynthesis [24]. Therefore, the present study was conducted to investigate the response to UV-B stress by using *R. chrysanthum* leaves as experimental material. Flavonoids belong to a group of phenolic compounds with antiviral activity; antioxidant, anti-inflammatory, antimutagenic, and anticancer properties; and an important role in ROS dynamic homeostasis [37,38,39]. Some studies have explored the relationship between the structure of flavonoids and the scavenging of hydroxyl radicals and concluded that flavonoids are potent scavengers of hydroxyl radicals and can be used as natural plant antioxidants [40]. Since *R. chrysanthum* produces a large amount of ROS after being subjected to abiotic stress, the property of flavonoids to regulate ROS homeostasis may enable *R. chrysanthum* to adapt to stressful environments [22]. In this study, we investigated the key enzyme genes affecting UV-B radiation resistance in *R. chrysanthum* and the transcription factors that may regulate the expression of these genes, using flavonoids as the main metabolic components.

The rETR is able to estimate PSII activity and photosynthetic efficiency, which reflects the rate of electron transfer driven by photosynthetically active radiation absorbed per unit of chlorophyll [41]. It can, therefore, monitor changes in the physiological state of plants under stress [42]. I_K_ can be used to assess the photosynthetic efficiency of plants under bright light and reflects the ability of a sample to tolerate bright light [43]. The higher its value, the more tolerant the sample is to bright light [44]. “1-qP” reflects the degree of reduction of QA (primary electron acceptor). Thus, “1-qP” measures the degree of center closure, i.e., the proportion of reaction centers that are not involved in the photochemical reactions of photosynthesis [45]. UV-B caused a significant decrease in rETR and “1-qP” and a significant increase in I_K_ in the experimental material. This shows that UV-B damages the photosynthetic system and reduces the resistance to strong light in this species. *R. chrysanthum*, therefore, mobilizes its own metabolic pathways to tolerate this adverse factor.

In this study, a total of 487 flavonoids were identified, 113 of which were significantly different between the M group and the N group, indicating that UV-B radiation significantly altered the accumulation of these flavonoids. These 113 flavonoids had the highest number of flavones and flavonols, suggesting that flavones and flavonols may be conducive to the acclimatization of *R. chrysanthum* to UV-B stress. Flavone is one of the largest flavonoids present in plants and can play a role in defending the plant against pathogen attacks and unfavorable environmental conditions such as salinity and high levels of UV-B exposure. It has been shown that apigenin and lignans in flavones are able to increase the tolerance of plants exposed to UV-B. Apigenin and its glycoside derivatives are able to protect plants from oxidative damage caused by UV-B radiation by reducing ROS production [46]. Baicalein contained in *Scutellaria* can act as an H_2_O_2_ scavenger to protect cells from oxidative stress [47]. Elevated levels of flavones (luteolin 7-o-glycosides and luteolin) and flavonols upon UV-B exposure represent a protective mechanism in *Artemisia annua* [18]. Flavonols have been shown to protect plants from damage to the photosynthetic electron transport mechanism and to be more important for protection against PSII damage than CO_2_ fixation. The induction of flavonol synthesis protects plants from UV-B stress, an effect that can be realized through their UV-absorbing properties (flavonols are capable of absorbing UV-B light at 280–320 nm) but also through the reduction of ROS once formed [48].

Some of the flavonoids detected in *R. chrysanthum* that produced significant differences under UV-B stress were not annotated in the KEGG database, suggesting that these flavonoids are not well studied in model plants and may be unique adaptive changes in *R. chrysanthum*. Isorhamnetin-3-o-neohesperidoside*, isorhamnetin-3-o-gallate, nepetin, limocitrin-3-o-arabinoside, cyanidin 3-xyloside, and other flavonoids, although not annotated in the KEGG database, were significantly elevated following UV-B radiation, suggesting that these substances can promote *R. chrysanthum*’s resistance to UV-B radiation. Twenty of the one hundred and thirteen significantly different flavonoids were able to be annotated to the KEGG database, so we focused on these twenty metabolites. RNA-seq data from the M and N groups showed 42 DEGs associated with flavonoid synthesis, significantly enriched in four pathways. Protein network interaction analysis of the DEGs revealed that TRINITY_DN8611_c0_g2_i3-C_2 has a direct interaction with TRINITY_DN8611_c0_g2_i1-C_2, both belonging to the genes encoding flavonol synthase, and the molecular functions showed that they have oxidoreductase activity. TRINITY_DN4856_c0_g5_i2-A_1 and TRINITY_DN21396_c0_g1_i1-C_3 have a direct interplay with CYP75A, capable of encoding flavonoid 3′,5′-hydroxylase with oxidoreductase activity, which acts on paired donors, and DFR, which can encode bifunctional dihydroflavonol 4-reductase/flavanone 4-reductase with dihydrokaempferol 4-reductase activity.

The correlation analyses of significantly different flavonoids with DEGs revealed that different metabolites had different correlations for the same gene and the same metabolite had different correlations for different genes. Among the four flavonoid-related pathways, the flavonoid biosynthesis pathway had the highest number of edges, with correlations between metabolites and DEGs greater than 0.9, suggesting that the screened DEGs may influence the synthesis of relevant metabolites in this pathway. This pathway had the most edges linked to naringenin chalcone, suggesting that naringenin chalcone may be an important metabolite in the response of *R. chrysanthum* to UV-B stress. One molecule of *p*-coumaroyl-CoA and three molecules of malonyl-CoA generate one molecule of naringenin chalcone, four molecules of CoA, and three molecules of CO_2_ by multi-step reaction in the presence of chalcone synthase. Naringenin chalcone as a node continues to generate flavonoids that play an important role in plant resistance, such as naringenin, dihydrokaempferol, cis-3,4-leucopelargonidin, and afzelechin. The chalcone isomerase-related genes in the transcriptomic data were TRINITY_DN92_c1_g1_i1-B_3 and TRINITY_DN92_c1_g1_i4-B_3 (expression as shown in the heat map in Figure 7), and naringenin chalcone was produced by the action of the chalcone isomerase naringenin.

In response to various abiotic stresses, the transcription of genes encoding enzymes of the flavonoid biosynthetic pathway is regulated by a combination of transcription factors [49]. It has been shown that the MYB, bHLH, WRKY, and NAC TF families are associated with flavonoid biosynthesis [50]. The MYB transcription factors influence the biosynthesis of proanthocyanidins, anthocyanidins, flavonols, and lignans in plants and play a key role in plant responses to adversity [51]. It has been shown that the MYB TFs are able to regulate the isoflavonoid biosynthesis pathway in soybean. For example, the R1-type MYB TF *GmMYB176* was able to regulate *CHS8* expression, affecting isoflavonoid synthesis [52,53]. bHLH is able to influence the composition of flavonoids in pomegranate [54]. Many bHLH genes have been detected in wheat, rice, and other crops involved in plant response to abiotic stresses [55]. bHLH plays an important role in plant adaptation to harsh environments by binding to specific sites on promoters to transcribe and regulate its target genes and direct plant metabolic processes [56]. Studies in grape have shown that the WRKY TF *VvWRKY26* is able to participate in flavonoid biosynthesis, which lays the foundation for studying the mechanism of flavonoid accumulation in grape [57]. WRKY is able to activate the transcription of antioxidant pathway genes, e.g., the overexpression of *MfWRKY70* in transgenic *Myrothamnus flabellifolia* was able to improve tolerance to drought and salt stress by regulating H_2_O_2_ and antioxidant levels, as well as by enhancing the transcription of genes related to stress response markers [58]. WRKY is able to regulate ROS homeostasis, thus playing a role in defense responses [59]. WRKY may affect the response of *R. chrysanthum* to UV-B radiation. NAC enhances plant survival and plant secondary growth under environmental stress conditions and plays a role in plant adaptation to stressful environments [60]. The transcriptomic data showed that more AP2-EREBP members were significantly different following UV-B stress and significantly differentiated flavonoids and DEGs were more highly correlated with AP2-EREBP, so AP2-EREBP members may be involved in the response of *R. chrysanthum* to UV-B stress by affecting gene expression.

Figure 7 demonstrates the upstream–downstream relationships of genes, TFs, and metabolites, revealing the regulatory mechanisms of flavonoid accumulation in *R. chrysanthum* under UV-B stress. This pathway involves a total of 13 significantly different flavonoids, 9 DEGs, and 7 potentially functioning WRKY TFs. These TFs are able to influence the expression of chalcone isomerase, dihydromyricetin reductase, flavonol synthase, and flavanoid 3′,5′-hydroxylase, thereby affecting downstream flavonoid synthesis. The specific regulatory mechanisms of TFs need to be further investigated.

## 4. Materials and Methods

### 4.1. Plant Materials and Treatment

Culture conditions and radiation treatments of experimental materials involved in this experiment followed previous experiments [61,62]. The experimental materials of this study were *R. chrysanthum* specimens collected from Changbai Mountain. After transferring *R. chrysanthum* from its native habitat to the laboratory, it was cultured in artificial climate chambers (daytime temperature of 18 °C, white light irradiation for 14 h, nighttime temperature of 16 °C, darkness for 10 h, and relative humidity 60%) by using 1/4 MS medium. After growing and culturing into 8-month-old seedlings, they were subjected to subsequent radiation treatments accordingly.

**Figure 8:**
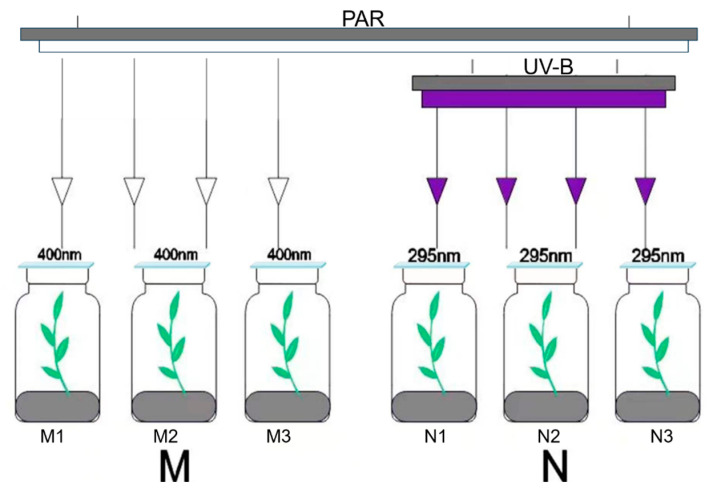
Schematic diagrams of the radiation treatments in the M and N groups.

The light conditions required to foster the growth of *R. chrysanthum* were imposed during radiation, with additional UV-B being applied to the N group. After being exposed to PAR radiation (400–700 nm), M group samples were put on a 400 nm long-pass filter (Filter Long 2IN SQ; Edmund, Barrington, NJ, USA). The N group used a 295 nm long-pass filter with PAR + UV-B (280–315 nm) irradiation (Figure 8). The filters were able to allow for the transmission of light of the corresponding wavelength. PAR was generated by using warm-white fluorescent lamps (T5 14W; Philips, Amsterdam, The Netherlands), while UV-B radiation was emitted by UV-B fluorescent lamps (Ultraviolet-B TL 20W/01 RS; Philips, Amsterdam, The Netherlands). According to the transmission characteristics of the long-pass filter, the actual photosynthetically active radiation and UV-B intensities for the plant materials measured 50 µmol(photon)/m^2^·s and 2.3 W/m^2^. For two days, the experimental therapy was administered eight hours a day. Three biological replicates were performed in each group. *R. chrysanthum* leaves were stored in liquid nitrogen. Lastly, transcriptomics and metabolomics were performed on these leaves.

### 4.2. Widely Targeted Metabolomics for Metabolite Identification and Quantification

Metware Biotech Inc., located in Wuhan, China, conducted a comprehensive metabolomic analysis for this study. The experimental methods and conditions adhered closely to those detailed in prior research [63].

Chromatographically pure grades of methyl alcohol, acetonitrile (Merck, Rahway, NJ, USA), and methanoic acid (Aladdin, Calhoun, GA, USA) were used for the standards. To extract metabolites from dry samples, the leaves were subjected to vacuum freeze drying and then pulverized into a fine powder by using a grinder. Subsequently, a 50 mg portion of the powdered sample was weighted and combined with 1200 μL of a pre-chilled (at −20 °C) 70% methanol aqueous solution containing an internal standard. We then performed vortexing 6 times (each lasting 30 s at 30 min intervals) and centrifuging (12,000 rpm for 3 min). A microporous filter membrane (0.22 μm) was used to filter the supernatant, which was then stored.

Metabolite separation and detection were performed by using ultraperformance liquid chromatography–tandem mass spectrometry (UPLC-MS/MS). The metabolites eluted during each interval were used to determine which set of multiple-reaction monitoring (MRM) ion pairs were monitored throughout each time. The MRM process is schematized in Figure S3. Based on the Metware database (MWDB), substance characterization was carried out based on secondary spectral information, and isotope signals, repetitive signals containing K^+^, Na^+^, and NH4^+^, were removed from the analysis. Repeated signals of fragment ions, which are themselves other substances of higher molecular weight, were removed. Triple quadrupole mass spectrometry was used for MRM analysis in order to quantify metabolites. To obtain metabolite-related data metrics, the mass spectra of the same metabolite in several samples were integrally corrected for outgoing peaks.

### 4.3. RNA-Seq Library Construction and Analysis

The RNA sequencing in this study was conducted by BGI Genomics Co., Ltd., Beijing, China, adhering closely to the methodologies and parameters outlined in prior research [64].

We extracted total RNA from the samples by using the CTAB procedure. Following this, single-strand circular DNA libraries were prepared and analyzed via RNA-seq.

The MGISEQ-2000 platform was employed for RNA-seq analysis. The initial step involved counting and filtering the raw sequencing data with SOAPnuke software (version 1.4.0) to eliminate sequences of poor quality. Subsequent sequence refinement was carried out by using trimmomatic to enhance data integrity. The clean reads were then mapped to the reference gene sequences by using Bowtie2 software (2.2.5), and the gene and transcript expression levels were quantified with RSEM (1.2.8). To identify changes in gene expression post-treatment, the FPKM method was applied to assess the expression levels of each transcript. The DESeq2 method, which is founded on the negative binomial distribution, was used to identify differentially expressed genes in *R. chrysanthum* following the treatments. Genes were considered differentially expressed if they had fold change (FC) > 1 and a q-value (adjusted *p*-value) < 0.05.

For the functional annotation and classification of unigenes, we utilized public databases such as KEGG, Pfam, and Swissprot. The annotation and classification process involved aligning Unigene sequences with those in the databases by using BLAST for comparison.

The transcriptome sequencing of *R. chrysanthum* was performed by the IlluminaHiSeq platform. The sequencing of the six samples yielded a total of 45.44 million raw reads each. After quality control measures, the range of clean reads obtained was between 42.21 million and 43.51 million. Additionally, the total clean bases ranged from 6.33 to 6.53 gigabases (Gb). These numbers show that the sequencing results were of a high caliber.

### 4.4. Measurement of Characteristics of Chlorophyll Fluorescence

The assay was based on the earlier experimental protocol [65]. The IMAGING-PAM chlorophyll fluorescence imaging system by Heinz Walz, Germany, was utilized to ascertain the PSII chlorophyll fluorescence parameters and compute associated parameters in the leaves of *R. chrysanthum*. The experiment took place in a dark setting with the ambient temperature kept consistent with the incubation temperature of the samples, ranging from 16 °C to 18 °C.

Prior to the commencement of the test, the leaves were allowed to adapt to darkness for a duration of 30 min. The minimal fluorescence level, denoted by Fo, was recorded under light-induced conditions. Subsequently, maximum fluorescence, known as Fm, was achieved through the application of saturating light pulses. Once the fluorescence intensity had returned to Fo, photochemically induced fluorescence kinetics were initiated at an intensity of 1600 μmol·m^−2^·s^−1^. Saturating pulses were applied every 20 s to ascertain both Fm′, which is the maximum fluorescence under light conditions, and the actual fluorescence intensity, F, at any given moment.

The rETR (relative electron transfer rate), 1-qP, and I_k_ were finally determined and calculated.

rETR = PAR·Y(II)·0.84·0.5

1-qP = 1-1-(F-Fo’)/(Fm’-Fo’)

I_k_ = rETR_max_/α (α: Initial slope of the relative electron transfer rate curve).

### 4.5. Statistical Analysis

The experiments were conducted three times by using a completely randomized design and analyzed by using IBM SPSS Statistics 26. One-way ANOVA was employed to assess the significance of the results. When significant differences were detected, Duncan’s test was applied to identify mean differences at the *p* < 0.05 level.

Figures in the metabolomics results section were plotted by using Metware Cloud (https://cloud.metware.cn). Metabolite correlation was processed by using Origin2022.

We used metaboanalyst5.0 for VIP value plots and boxplots. A variable importance in projection (VIP) plot, which is commonly used in PLS-DA, was used for ranking the metabolites based on their importance in discrimination between the M group and the N group *R. chrysanthum*. VIP score is a weighted sum of squares of the PLS loadings. The amount of explained Y-variance in each dimension influenced the weights [66].

The Orthogonal Partial Least Squares-Discriminant Analysis (OPLS-DA) model was established by utilizing the MetaboAnalystR R package, version 1.0.1, following the log2 transformation and subsequent centering of the raw data.

Transcriptomic data were analyzed by using the Dr. Tom platform. Correlations between differentially significant metabolites obtained from metabolomic analysis and DEGs obtained RNA-seq were analyzed by using Pearson correlation analysis with a correlation analysis threshold of 0.9 and *p* < 0.05. Correlation network mapping was performed by using Cytoscape 3.8.0.

## 5. Conclusions

In the present study, the response to UV-B radiation was investigated by using *R. chrysanthum* group seedlings grown in artificial climate chambers as the experimental material. In this study, we investigated the metabolic changes in the leaves of *R. chrysanthum* following UV-B radiation by using widely targeted metabolomics and transcriptomics. By screening, we obtained 13 significantly different flavonoids, 9 DEGs, and 7 potentially functioning WRKY TFs of the flavonoid-related synthetic pathway. The results show that WRKY TFs could affect the expression of chalcone isomerase, dihydromyricetin reductase, flavonol synthase, and flavonoid 3′,5′-hydroxylase under UV-B stress, thus affecting the flavonoid content. However, the specific mechanisms by which TFs regulate gene expression need to be further investigated. This study holds significant practical importance for understanding the dynamics of metabolite changes and the regulatory mechanisms of plant growth adaptation under UV-B stress conditions. The findings are instrumental in forecasting the future impacts of increased UV-B radiation due to ozone depletion on plant communities and ecosystems.

## Figures and Tables

**Table 1:** Information on genes encoding key enzymes in the flavonoid pathway.

Name	Enzyme	Gene ID	log2 (N/M)	Q-Value (M vs. N)
Flavonoid 3′,5′-hydroxylase	1.14.14.81	TRINITY_DN15393_c0_g2_i3-A_2	−2.051	0.002
		TRINITY_DN4856_c0_g5_i2-A_1	−2.614	0.000
Chalcone isomerase	5.5.1.6	TRINITY_DN92_c1_g1_i1-B_3	1.074	0.002
		TRINITY_DN92_c1_g1_i4-B_3	1.303	0.000
Dihydromyricetin reductase	1.1.1.219	TRINITY_DN21396_c0_g1_i1-C_3	1.967	0.001
Flavonol synthase	1.14.20.6	TRINITY_DN1689_c0_g1_i1-A_2	−1.588	0.010
		TRINITY_DN6320_c0_g1_i1-C_1	−1.258	0.000
		TRINITY_DN8611_c0_g2_i1-C_2	−1.468	0.028
		TRINITY_DN8611_c0_g2_i3-C_2	−1.155	0.022

## Data Availability

The data used in this study are available from the corresponding author upon submission of a reasonable request.

## Supplementary Materials

The following supporting information can be downloaded at: https://www.mdpi.com/article/10.3390/plants14010133/s1, Table S1: Information on metabolites identified by widely targeted metabolomics, Table S2: Differentially expressed genes associated with flavonoid synthesis, Table S3: Correlation analysis of WRKY TFs with key enzyme genes in flavonoid synthesis pathway, Figure S1: Metabolomic changes under stress, Figure S2: DEG KEGG analysis and GO analysis, Figure S3: Schematic diagram of multiple-reaction monitoring (MRM) process.

## Abbreviations

*Rhododendron chrysanthum* Pall.	*R. chrysanthum*
differentially expressed genes	DEGs
fold change	FC
Kyoto Encyclopedia of Genes and Genomes	KEGG
Orthogonal Partial Least Squares-Discriminant Analysis	OPLS-DA
photosynthetically active radiation	PAR
photosystem II	PSII
primary electron acceptor	QA
relative electron transfer rate	rETR
transcription factor	TF
ultraviolet-B	UV-B
UV RESISTANCE LOCUS 8	UVR8
variable importance in projection	VIP
