# Supplementary material for: WRKY Transcription Factors Modulate the Flavonoid Pathway of Rhododendron chrysanthum Pall. Under UV-B Stress
Source: Plants (Basel). 2025 Jan 4;14(1):133. doi: 10.3390/plants14010133 (PMC11723172; doi:10.3390/plants14010133)
Supplement: Supplementary file 1 [file plants-14-00133-s001.zip › Supplementary Figuers .pdf]

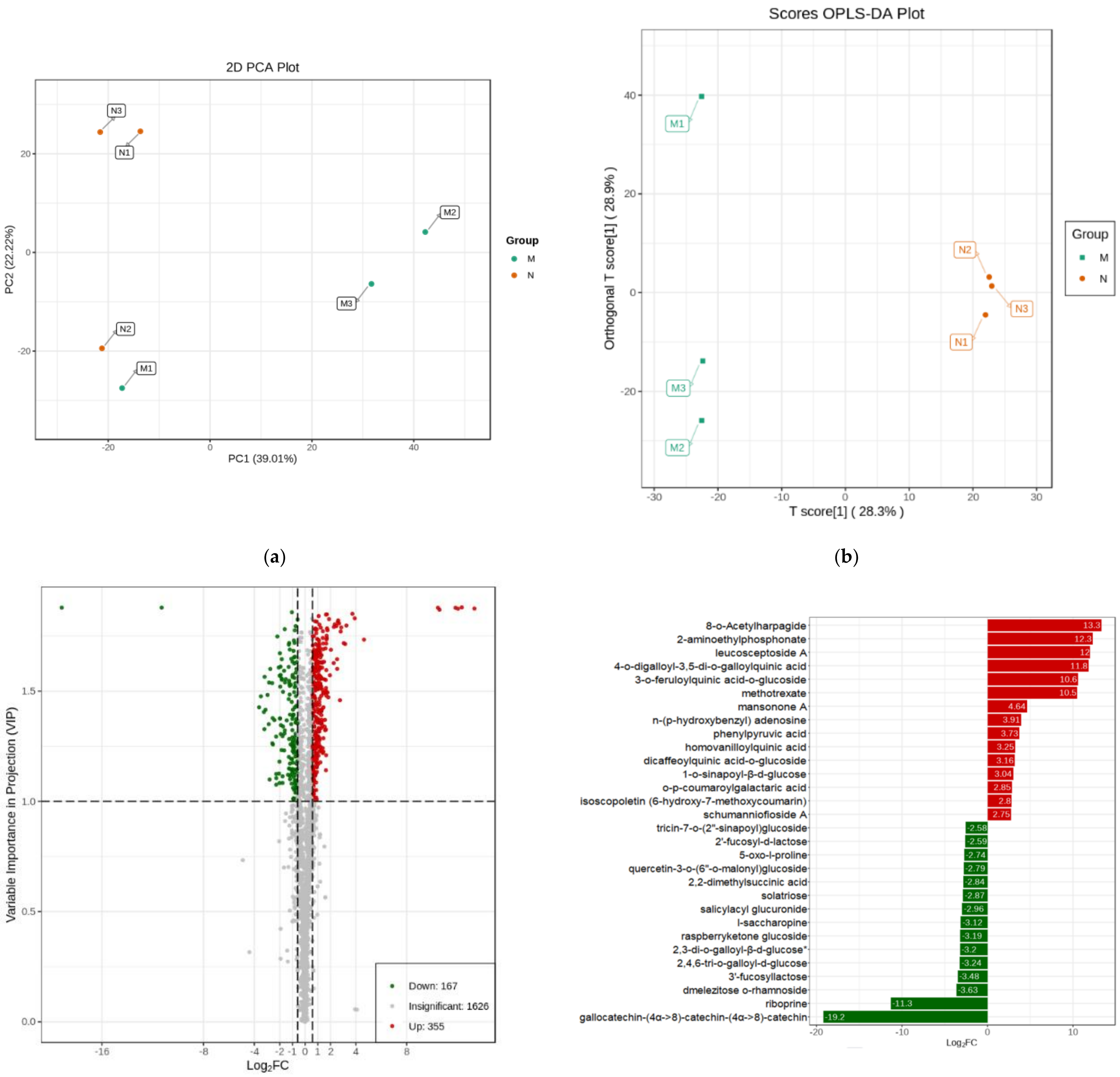
2D PCA Plot
PC1 (39.01%)
PC2 (22.22%)
N3
N1
M2
M3
N2
M1
Group
M
N
Scores OPLS-DA Plot
T score[1] (28.3%)
Orthogonal T score[1] (28.9%)
Log2FC
Variable Importance in Projection (VIP)
Down: 167
Insignificant: 1626
Up: 355
8-o-Acetylharpagide 13.3
2-aminoethylphosphonate 12.3
leucosceptoside A 12
4-o-digalloyl-3,5-di-o-galloylquinic acid 11.8
3-o-feruloylquinic acid-o-glucoside 10.6
methotrexate 10.5
mansonone A 4.64
n-(p-hydroxybenzyl) adenosine 3.91
phenylpyruvic acid 3.73
homovanilloylquinic acid 3.25
dicaffeoylquinic acid-o-glucoside 3.16
1-o-sinapoyl-β-d-glucose 3.04
o-p-coumaroylgalactaric acid 2.85
isoscopoletin (6-hydroxy-7-methoxycoumarin) 2.8
schumanniofioside A 2.75
tricin-7-o-(2''-sinapoyl)glucoside -2.58
2'-fucosyl-d-lactose -2.59
5-oxo-l-proline -2.74
quercetin-3-o-(6''-o-malonyl)glucoside -2.79
2,2-dimethylsuccinic acid -2.84
solatriose -2.87
salicylacyl glucuronide -2.96
l-saccharopine -3.12
raspberryketone glucoside -3.19
2,3-di-o-galloyl-β-d-glucose* -3.2
2,4,6-tri-o-galloyl-d-glucose -3.24
3'-fucosyllactose -3.48
dmelezitose o-rhamnoside -3.63
riboprine -11.3
gallocatechin-(4α->8)-catechin-(4α->8)-catechin -19.2
Log2FC

(**a**) (**b**)

(**c**) (**d**)

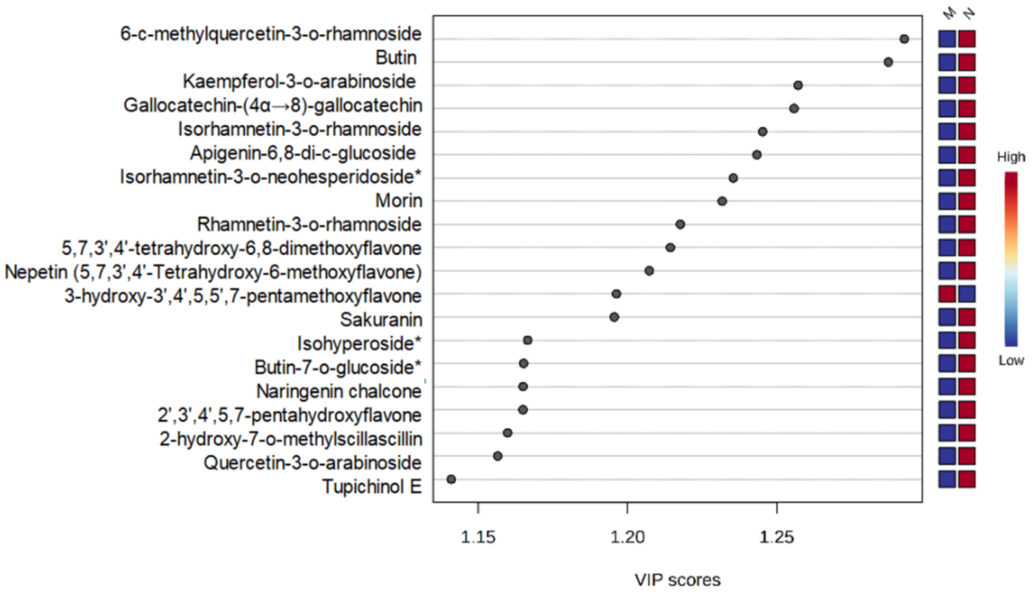

(e)

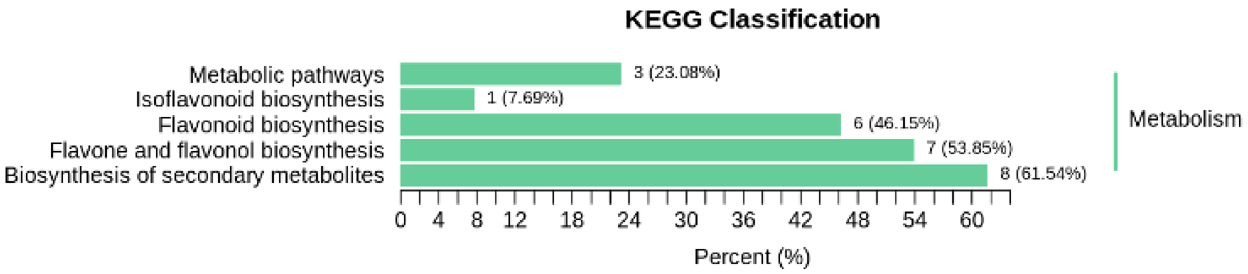

(f)

Figure S1. Metabolomic changes in *R. chrysanthum* after UV-B stress. **(a)** PCA analysis; **(b)** OPLS-DA analysis; **(c)** Volcano map; **(d)** The first 30 FC of the differential metabolites were mapped; **(e)** VIP plot: important features (metabolites) identified by PLS-DA in a descending order of importance; **(f)** KEGG enrichment analysis bar chart of flavonoids. In the volcano and multiplicity-of-difference bar graphs, metabolites with increased contents are shown in red and metabolites with decreased contents are shown in green. Figure S1e represents relative contribution of metabolites to the variance between M group and N group *R. chrysanthum*. High value of VIP score indicates great contribution of the metabolites to the group separation. The red and blue boxes on the right indicate whether the metabolite content is increased (red) or decreased (blue) in the leaves of M group and N group *R. chrysanthum*. Blue to red colors in the color bar indicate low to high metabolite levels. The y-axis in Figure S1f represents the peak area of metabolites in the sample obtained by widely targeted metabolomics assays.

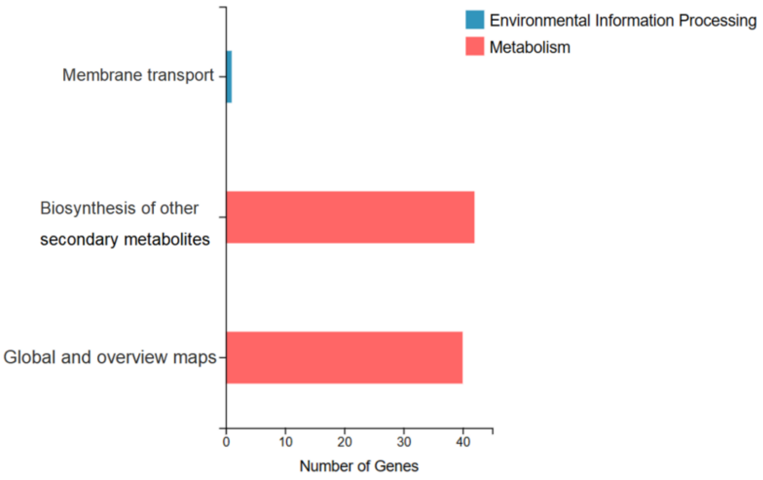

(a)

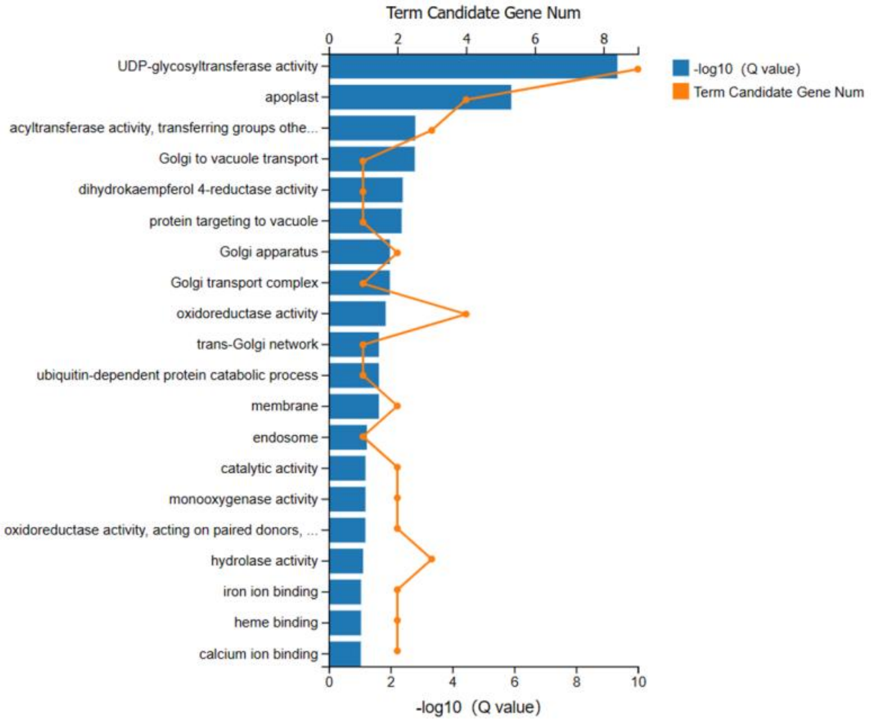

(b)

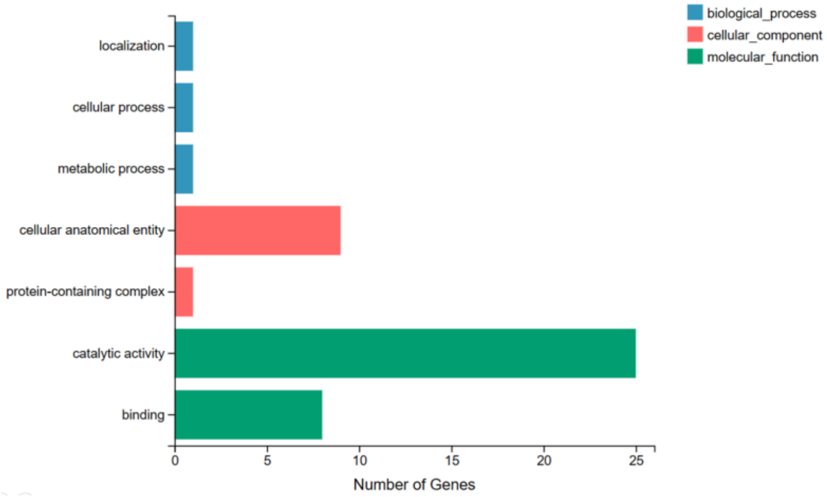

(c)

Figure S2. Differentially expressed genes KEGG analysis and GO analysis. **(a)** KEGG enrichment analysis bar chart; **(b)** GO Enrichment; **(c)** GO Classification.

Metabolite quantification was accomplished using triple quadrupole mass spectrometry in multiple reaction monitoring mode (MRM, Figure S3) analysis. In MRM mode, the four-stage rod firstly screens the precursor ions (parent ions) of the target substances, and excludes the ions corresponding to other molecular weight substances to eliminate the interference initially; the precursor ions break to form a lot of fragment ions after induced ionization by the collision chamber, and the fragment ions are filtered through the triple four-stage rod to select a characteristic fragment ion required, which excludes the interference of the non-target ions, and makes the quantification more accurate and reproducibility better. After obtaining the metabolite profiling data of different samples, the peak area integrations of all the substance peaks were performed, and the integrals were corrected for the mass spectrometry peaks of the same metabolite in different samples.

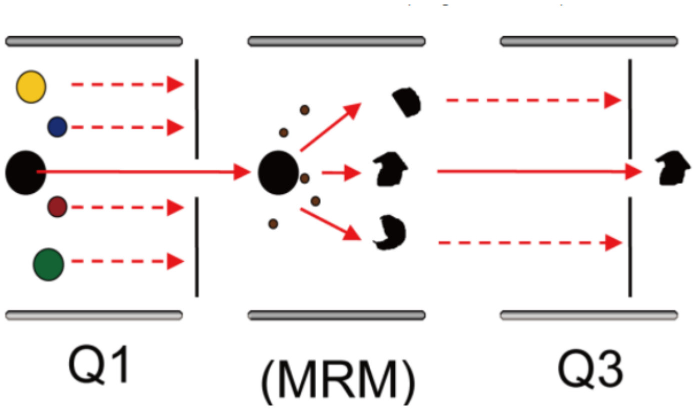

Figure S3. Schematic diagram of the multiple reaction monitoring (MRM) process.
